# Near-Infrared Spectroscopy for Growth Estimation of *Spirulina platensis* Cultures

**DOI:** 10.3390/mps7060091

**Published:** 2024-11-03

**Authors:** Lamprini Malletzidou, Eleni Kyratzopoulou, Nikoletta Kyzaki, Evangelos Nerantzis, Nikolaos A. Kazakis

**Affiliations:** Laboratory of Archaeometry and Physicochemical Measurements, Athena- Research and Innovation Center in Information, Communication and Knowledge Technologies, Kimmeria University Campus, PO Box 159, GR-67100 Xanthi, Greece; e.kiratzopoulou@athenarc.gr (E.K.); nikoletta.kyzaki@athenarc.gr (N.K.); e.nerantzis@athenarc.gr (E.N.); nikkazak@athenarc.gr (N.A.K.)

**Keywords:** microalgae, cultivation, NIR, PCA, PLS, Spirulina medium

## Abstract

The present study proposes the use of Near-Infrared (NIR) spectroscopy as a rapid method for estimating the growth of *Spirulina platensis* cultures, avoiding any sample manipulation or pretreatment. NIR spectroscopy in diffuse reflectance mode was used on culture volumes as received, with Principal Component Analysis (PCA) and Partial Least Squares (PLS) linear regression, for developing the calibration model in the wavelength range of 1000–2500 nm, in order to choose the appropriate wavelength to estimate the growth of the microalga. The local reflectance maximum at 1062.6 nm, connected with reduced water absorption and scattering effects by the microalga, was identified from PCA as the positive peak in the first loading plot, correlating diffuse reflectance with dilution levels. The calibration curve of diffuse reflectance at 1062.6 nm in response to dilution presented strong linearity, supported by a coefficient of determination (R^2^) of 0.995. Cross-validation of NIR spectra with a *S. platensis* culture confirmed the method’s reliability, showing that the growth follows an exponential pattern. The study shows that diffuse reflectance NIR spectroscopy can be used for the rapid monitoring of *Spirulina platensis* growth.

## 1. Introduction

Microalgae cultivation and harvesting have drawn interest because of their various applications in pharmaceutics [1], food supplements [1,2], production of chemical compounds [1,3], biofuel applications [3,4,5], fertilizers [6], and removal of heavy metals [7,8,9]. Considering their faster growth rate and lower volumes of growth area requirements in comparison to terrestrial plants [3], microalgae are also regarded as industrially and domestically significant enough to be cultivated.

*Spirulina platensis* (*S. platensis*)—previously grouped in the genus *Arthrospira*—is a marine filamentous cyanobacterium [2], used as a food supplement not only in human food but also in the animal feed industry [10], because of its high protein level [2,11,12], and its content of vitamins, amino acids, fatty acids, glycolipids, sulfolipids, and minerals [1,2,13,14]. Moreover, its pharmaceutical applications are recognized due to its content in anti-viral, anti-bacterial, and anti-tumor agents, and anti-oxidants [2], together with its use for chlorophyll [15] and phycocyanin [16] production. Additionally, its use for bioremediation is also explored, for the removal of heavy metals [7,17,18,19], and recycling of lignite fly ash [20], without ignoring its application for the production of biofuels [21]. Its popularity also originates from its use as a food supplement together with its ability to be domestically cultivated.

Considering the various applications of microalgae, it is crucial to estimate their biomass or population, for basic research, industrial and ecological reasons [22], which is also highlighted by the reviews that deal with the subject of microalgae biomass and/or estimation [10,23,24,25]. Several methods have been employed in this direction, with advantages and limitations in every case as follows:

- Cell counting by haemocytometry. Defined culture volumes are placed on a haemocytometer with a cover glass on top, and the number of existing microalgae cells are directly and manually counted using a microscope. Although this method allows the distinction between live or non-viable cells, it is time consuming, and the drawbacks in the case of agglomerations and human error are noted. In the case of *S. platensis*, these problems are intensified because of its variable length [10,26]; the filaments usually vary between 50 and 500 μm [27]. Nevertheless, direct counting with a microscope using a 10× objective has been reported [20].
- Dry weight measurement. Defined culture volumes are centrifuged to remove the culture medium, or filtered, dried, and weighted [2]. In the case of *S. platensis*, the step of washing with distilled water is introduced, to remove the inorganic salts of the culture medium [1,2,16,28,29,30,31]. Although this method has high accuracy, it is quite time consuming, and its requirements of large culture volumes, that cannot be returned to the culture, are noted [10].
- Optical density (OD) measurements: With OD, the light absorption of culture volumes at certain wavelengths in the UV-Vis region is reported [2,8,17,32], or with nephelometry [10]. Regarding *S. platensis*, OD measurements performed by UV-Vis spectrophotometry are reported at 560 nm [1,2,15,17,20,33], 600 nm [31], and 680 nm [16,29,34].
- Protein, lipid, carbohydrate, chlorophyl, and carotenoid content estimation. Such procedures require the extraction of these contents using solvents, or by fluorescence measurements that can also be performed in vivo [6,10]. The application of Fourier transform infrared (FTIR) spectroscopy for this quantitative analysis is also reported [22]. Regarding chlorophyl, its content estimation leads to an indirect cell growth estimation [13], which also acts as a qualitative estimation of the culture itself. This leads to considerations when chlorophyll is not consistent, i.e., when culture conditions are not stable or do not promote chlorophyll production. Apart from the use of solvents for calculating the aforementioned contents, the use of direct measurements using spectroscopic techniques (i.e., attenuated total reflectance Fourier transform infrared spectroscopy, ATR-FTIR, and Raman spectroscopy) are mentioned, for the estimation of carbohydrates of microalgae in general [10], and the glucose and protein contents of *S. platensis* [35], without any extraction.
- Hyperspectral imaging or other image processing methods. Image processing methods [36,37], often employing machine learning and data analysis, are gaining interest for their ability to handle big data, even obtained from online measurements [10,26]

Based on the above and considering the growing interest in *S. platensis* cultivation on a domestic, laboratory, and industrial scale, there is a need for developing new, fast and reliable methods to estimate its mass and/or growth rate.

To this respect, Near-Infrared (NIR) spectroscopy could be an excellent method for such a purpose. NIR spectroscopy uses the near-infrared region of the electromagnetic spectrum (800–2500 nm) for the analysis of materials [38]. This spectral region is not directly related to fundamental vibrations, but to overtone bands and combinations of fundamental bands [38,39], resulting in complex spectra, more difficult to be interpreted compared to those of the mid IR region. Nevertheless, the method can provide non-destructive measurements, presenting advantages in measuring large quantities of samples, and it is easy to be applied [40]. Moreover, it can be evolved to allow in situ real-time measurements [23,41], with minimal sample or no sample preparation even regarding aqueous samples [42]. Furthermore, NIR spectroscopy can be used for developing accurate prediction models for quantification analysis [42]; NIR has been extensively used for chemometrics in a great variety of materials, from solids and powders to aqueous dilutions or dispersions, i.e., aqueous acid content [43,44], sugars of fruit juices [45], and fruits [46,47].

From all the spectroscopic techniques that are employed for microalgae analysis, NIR spectroscopy has been proposed as a way to monitor cell cultures [38] and microalgae cultures online [41]. So far, it has been used in the field of microalgae monitoring, in terms of composition analysis [39], especially in the case of dry biomass, to avoid diffuse reflectance phenomena [14,42,48,49,50,51,52]. The application of NIR, together with other techniques, such as FTIR spectroscopy, for online estimation of microalgae growth, is also investigated [10]. In the case of aqueous samples of microalgae, the lipid and biomass estimation using reflectance NIR with a probe is reported, too [53].

Microalgae cultures correspond to liquid samples with suspended material, which are better examined with transmittance or reflectance measurements under continuous stirring to ensure the sample’s homogeneity during measurement [54]. Similarly, the study of aqueous cultures by means of NIR spectroscopy is applicable, since water is considered a solvent for NIR, because of its very low interference in this spectral region [41]. In diffuse reflectance mode, the phenomena of absorbance and scattering are combined when light interacts with the sample, as this mode emerges from the refraction and reflection of light on the particles, resulting in unique spectral characteristics for every substance [41,55].

Based on the above, the present study focuses on examining the application of NIR spectroscopy as a reliable, fast and non-destructive technique for the biomass growth estimation of *S. platensis*, which could also be used for online measurements [24,38]. For this purpose, *S. platensis* was axenically cultivated in a laboratory using Spirulina medium and proper aeration and light conditions, to examine its NIR spectra in diffuse reflectance mode (DR-NIR). To choose the appropriate wavelength for the calibration curves, and to correlate the DR-NIR spectral response with the growth determination, Principal Component Analysis (PCA) and Partial Least Squares (PLS) linear regression were used [56]. These chemometric methods were applied to determine the appropriate wavelength of analysis, which linearly connects diffuse reflectance spectral response to dilution levels of culture. For validating the proposed method, experiments were performed on an axenically laboratory cultivated large scale (30 L open-type tank) *S. platensis* culture by checking its growth pattern.

## 2. Materials and Methods

**Figure 1:**
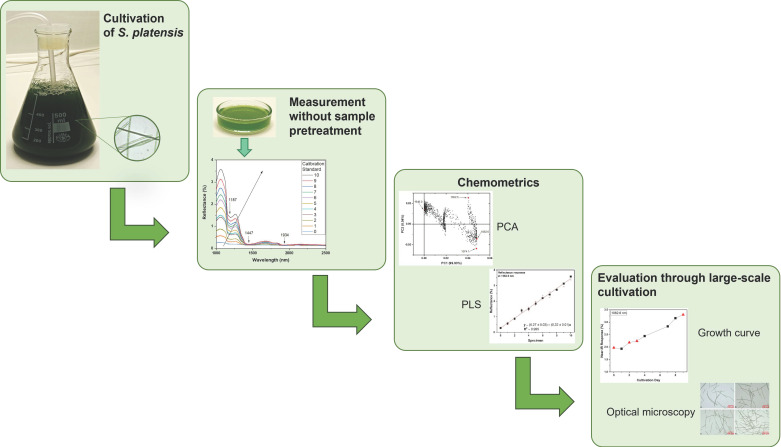
Schematic outline of present study.

The present study consists of four main steps, as presented in Figure 1 and described in the following subsections.

### 2.1. Cultivation of S. platensis

Fresh and living *S. platensis* cyanobacteria were procured by HEALTHALGAE Sweden AB.

Spirulina Medium (SM) was used as the growth medium for the cultivation of the cyanobacterium [10,57,58]. In brief, 1000 mL of SM are composed of four Stock Solutions (SLs), as presented in Table 1 (source of components: AnalytiChem Belgium NV/former Chem-Lab NV, Carl Roth, Sigma-Aldrich, and ACS Chemicals). Stock Solutions I and II were of a total volume of 500 mL each. Stock Solutions III and IV are characterized as micronutrient solutions. For clarity reasons, SL III is composed of individual solutions, where each micronutrient is prepared according to the indicated concentration [57]. After their preparation, SL I and SL II were autoclaved, mixed after cooling down to room temperature, and then the produced SM was stored at 4 °C. Finally, SM was used for *S. platensis* cultivation after reaching room temperature. Further details can be found in the cited literature [57].

*S. platensis* was axenically cultivated in laboratory conditions [1,17,30,35,59]. For this reason, 50 mL of the commercially procured cyanobacteria start culture were inserted in a 500 mL Erlenmeyer glass flask, inoculated to 450 mL of Spirulina Medium. The cultivation conditions were as follows: 27.0 ± 0.5 °C temperature, pH 9.4, under constant aeration (~1 L/min), and illumination was provided with a full spectrum LED array light source (LUMATEK, ATTIS 200W) with irradiance of ~160 μmol m^−2^ s^−1^ and a photoperiod of 12 h light:12 h dark. After one week of cultivation, aeration was stopped for two hours, and 100 mL of dense *S. platensis* culture was extracted from the bottom of the Erlenmeyer glass flask.

### 2.2. Preparation of Calibration Set

Primary standards could not be used for making the calibration curve relating *S. platensis* biomass to spectral response. For this reason, a modified serial dilution protocol was applied, that was based on standard addition protocol [60]. The dense *S. platensis* culture, that was received as described in Section 2.1, was used as the stock solution to prepare eleven dilution levels that were used as the calibration standards for the calibration curve. These dilution levels were prepared mixing volumes of the dense *S. platensis* culture with Spirulina Medium, as presented in Table 2. It should be mentioned that plain Spirulina Medium was employed as the calibration standard #0, while the dense *S. platensis* culture was the calibration standard #10.

### 2.3. Near-Infrared Spectroscopy

The measurements were performed with a NIR spectrometer, model SPECTRUM Two N™, PerkinElmer, with a tungsten-halogen source and an InGaAs detector, equipped with a Near-Infrared Reflectance Module (NIRM) top plate and a diffused silica window, which allows diffuse reflectance analysis by directly placing containers of samples on top of the window.

Measurements were performed placing 10 mL of each sample (calibration standard, as described in Table 2) in a glass Petri dish of 60 mm diameter, without any other pretreatment besides its gentle stirring (at 300 rpm, for 5 s) using a ZX3 Advanced Vortex Mixer, VELP Scientifica (Italy) in the following two stages: just before sampling from the culture to ensure the homogeneity of the samples, because of sedimentation reasons, and after placing them inside the Petri dish just before the measurement. The spectra were collected by directly placing the Petri dish on the NIRM top plate with the following conditions: 8 cm^−1^ resolution with 64 accumulative scans. Measurements were performed in the spectral area of 1000–2500 nm (10,000–4000 cm^−1^). For each dilution level, duplicate measurements were performed to check the reproducibility of the collected spectra. Raw data were exported and used for analysis without any previous manipulation or spectral pretreatment, such as smoothing, normalization, or baseline correction. The chosen measurement conditions with −64 scans of 8 cm^−1^ resolution between 1000 and 2500 nm- have a duration of ~140 s. To check the microalgae sedimentation during this time period [31,61], which can lead to errors regarding their spectral response, another set of measurements was also performed. For each dilution level (Table 2), the same sample after the normal measurement, as described above, was again gently stirred, and seven successive DR-NIR spectra were recorded, this time with 6 scans, the same resolution and in the same spectral region, resulting in seven successive measurements of ~20 s duration each. The difference in the spectral response between the 1st (duration ~20 s) and the 7th (duration ~140 s) measurements can be considered as a systematic error [60], which was introduced to the PLS linear regression analysis.

### 2.4. PCA and PLS Linear Regression Analysis

The dataset of the calibration set consisted of 11 DR-NIR spectra of different dilution levels of *S. platensis*, that were collected as described in the previous subsections. The dataset was validated with Principal Component Analysis (PCA) and Partial Least Squares (PLS) regression using the STATISTICA (version 7) software, StatSoft, Inc., Tulsa, OK, USA, and plotted with the ORIGIN (version 7.5) software, OriginLab Corporation, Northampton, MA [62].

As the first step of analysis, PCA was performed to the dataset to identify the appropriate wavelength that showed the greatest variance of the spectra because of the dilution level. As previously stated, no spectral pretreatment was applied to the dataset prior to the analysis. Next, the reflectance values of the dataset at the wavenumber that was identified through PCA were used to examine the linearity of the reflectance response of the dataset to the different dilution levels. For this step, the systematic error that was introduced because of sedimentation of the microalga during the measurement (as described in Section 2.3) was taken into consideration. The linearity of the model was evaluated by the coefficient of determination (R^2^). It should be noted that the consistency of the measurements was checked in terms of reproducibility of the collected spectra, and no separate validation set was used. Following this, to evaluate the consistency of the model, the root mean squared error (RMSE) was recorded [63].

### 2.5. Validation

Calibration curve results were validated with a different *S. platensis* culture, axenically and laboratory cultivated on a larger scale. More specifically, 1.5 kg of wet biomass of *S. platensis*, obtained by centrifugation, was inoculated to freshly prepared SM, to obtain an initial ~50 g/L culture (wet biomass per SM liter) concentration in a 30 L open-type tank. For this reason, a X1R Pro centrifuge, Thermo Scientific™ (USA), was employed at 4000 rpm for 5 m. The culture was then cultivated for 9 days, under the same illumination, pH, temperature, and aeriation conditions, as described in Section 2.1. Samples were collected daily from the large-scale *S. platensis* culture and their DR-NIR spectra were recorded, with the same conditions that were applied for the calibration set, as described in Section 2.3.

Furthermore, the 30 L open-type tank culture was observed daily by means of optical microscopy, through all nine days of cultivation, to monitor the microalga growth and any morphological changes in the filaments [10,17]. For this reason, 30 μL of culture -after gentle stirring- were placed on a glass slide and shielded with a cover glass [17,31]. The samples were observed with an Olympus CX43RF microscope, equipped with an Olympus EP50 digital camera, using a 10× objective lens.

## 3. Results

### 3.1. Diffuse Reflectance Near-Infrared Analysis of S. platensis

**Figure 2:**
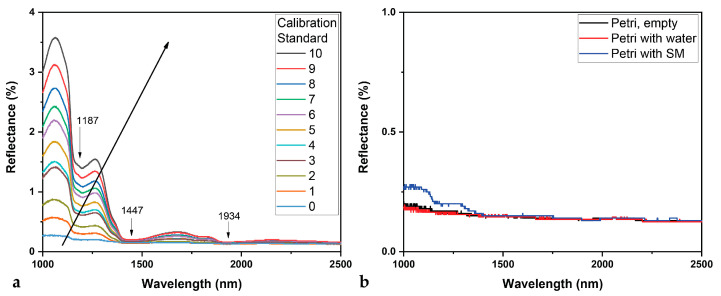
The DR-NIR spectra: (**a**) of all the calibration standard set (Table 2), where the arrow indicates the increase in the microalgae biomass; (**b**) of the Petri dish used for measurements empty, with distilled water, and with Spirulina Medium.

Figure 2a shows the DR-NIR spectra of all the calibration set of the microalga (Table 2). The collected spectra of *S. platensis* calibration set present strong absorbance bands in spectral positions that follow the absorbance spectra of plain water. This has also been reported in the transmittance NIR spectra of protein solutions [55], and the fat and protein content of milk [64]. In particular, the absorbance bands at 978 nm (10,224 cm^−1^), 1187 nm (8424 cm^−1^), 1447 nm (6910 cm^−1^), and 1934 nm (5170 cm^−1^) (see Figure 2a) are attributed to different combination modes of water, or to the binding of two water molecules [65]. A similar response has reported by Brown et al. [53], who deal with reflectance spectra of filtered microalgae. It is not safe to attribute the observed reflectance bands to any functional groups, given the fact that diffuse reflectance measurements result to very wide bands, and considering that NIR bands are also characterized by broadness, due to overlapping, resulting from the overtones and combination bands [55]. For this reason, although the reflectance bands of 1100–1200, 1350–1450, and 1650–1850 nm are attributed to lipids, as is the area of 1460–1570 nm to proteins [53], it is appropriate to deal with the absorbance bands of water, avoiding any other kind of characterization in terms of attribution of bands to functional groups [41]. This can be further supported by other examples in the literature regarding reflectance measurements of wet or aqueous samples that have similar NIR spectral profiles, such as in the case of mussel tissues as received [66].

Figure 2b presents the DR-NIR spectra of the Petri dishes that were used for the culture measurements, empty, with 10 mL of distilled water, and with 10 mL of SM. All measurements introduce a constant baseline shifting of 0.14%, while the SM spectral response does not exceed 0.27%. It is noted that water presents a small interference level to the DR-NIR measurements compared to other spectroscopic techniques.

### 3.2. PCA and PLS Linear Regression

The application of chemometric methods is essential to define the components that depict the spectral differences of interest [41]. Τhe bands at ~1060 and 1260 nm of the DR-NIR spectra of *S. platensis* calibration set (Figure 2a) are strong candidates to be selected for the calibration curve of reflectance spectral response versus dilution. This is because they mostly reflect these spectral changes, due to the dilutions using SM, and their higher signal-to-noise ratio. In order to select the appropriate wavelength for generating the calibration curve, PCA was performed to the collected DR-NIR spectra, to demonstrate which wavelength varies the most because of the dilution level.

**Figure 3:**
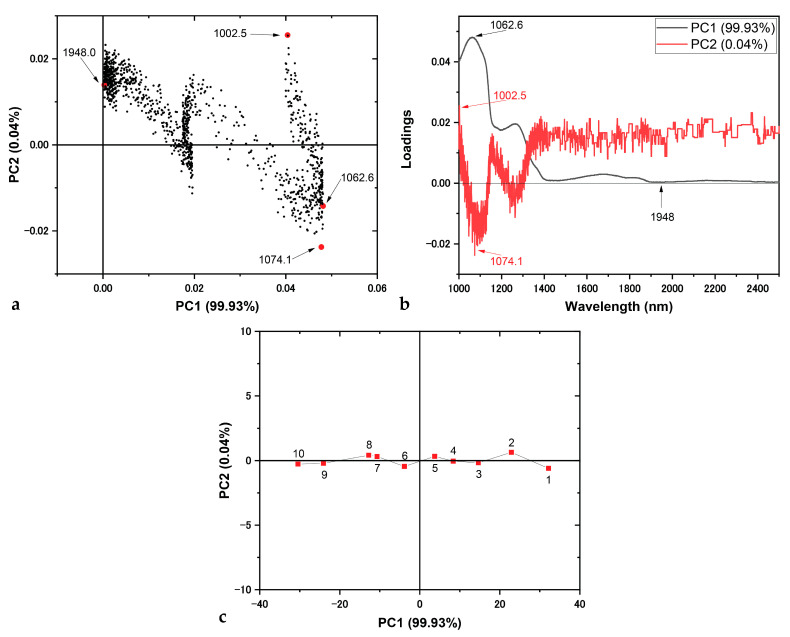
PCA performed on calibration dataset of *S. platensis*: (**a**) loading plot of PC1 vs. PC2; (**b**) loading plots of PC1 and PC2 vs. wavelength; (**c**) score plot. Numbers indicate the calibration standard (Table 2).

The PCA is visualized by the plots in Figure 3, illustrating which wavelength better influences the variance of the dataset. Figure 3a shows the scores plot of the first two principal components (PCs), i.e., the eigenvectors that define the directions of the PCs in the space that is defined by the data. The first principal component (PC1) explains the majority of the variance, which gathers 99.93% of the variations in the collected spectra, while the second (PC2) explains the 0.04%; the first two PCs together explain the 99.98% variation in the collected spectra. This shows that PC1 is enough to explain the variation between the collected spectra, while the variance inserted by PC2 is essentially null. At this point, it should be stated that these results were expected, as the only variation between the collected spectra is their dilution level. Thus, the one and only variance of the system is the dilution level itself, and this variation can be explained by one PC, while the linear combination of the variables, i.e., the diffuse reflectance spectra, is enough to explain more than 99% of the variations. Regarding PC1, that corresponds to the main reason of variability between the spectra of the calibration dataset, its highest eigenvector, with a score of 0.048, corresponds to the predicted band at ~1060 nm, and specifically to 1062.6 nm. This confirms that this band can be used for the calibration plot of *S. platensis* culture growth rate estimation. The highest eigenvector of PC2, with a score of 0.026, is located at 1002.5 nm, confirming its attribution to noise.

These results are better visualized by the loading plots of PC1 and PC2 versus wavelength in Figure 3b; the highest positive loading of PC1 is the 1062.6 nm wavelength. The graph also supports that PC2 is attributed to noise. Finally, the score plot of PC1 vs. PC2 is presented in Figure 3c. PC1 clearly shows that the distribution of the collected spectra is due to their dilution, while PC2 inserts small variations because of noise.

**Figure 4:**
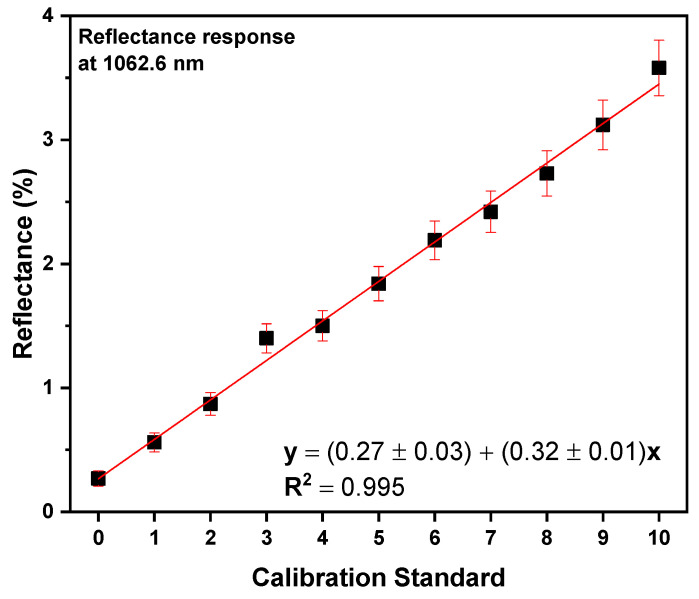
Scatter plot of DR-NIR at 1062.6 nm versus *S. platensis* dilution level, as presented in Table 2.

Figure 4 shows the reflectance spectral response of the 1062.6 nm wavelength that was derived by means of PCA versus the dilutions as presented in Table 2. The error bars regarding the reflectance spectral response derive from the sedimentation of *S. platensis* in the Petri dish that was used for the measurements during the measuring time. By applying PLS linear regression, it is shown that there is indeed a linear response between reflectance at 1062.6 nm and dilution level, with a value of 0.995 for the coefficient of determination (R^2^), and an RMSE value of 0.077 for the linear fitting.

The analysis, as expressed by the high R^2^ value, shows that the reflectance spectral response at 1062.6 nm has a linear relationship with the *S. platensis* biomass content, with comparable R^2^ values to other studies employing NIR spectroscopy for quantification reasons [40]. In addition, the low RMSE value indicates a low prediction error by the linear model.

### 3.3. Validation of Laboratory-Scale Results

**Figure 5:**
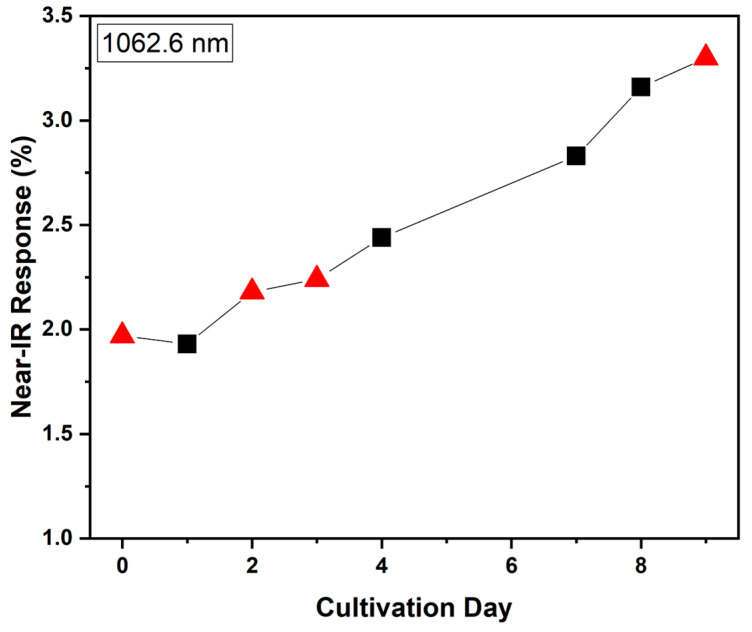
The biomass growth estimation curve of *S. platensis* cultivation in a 30 L open-type tank for 9 days. The data points depicted with red arrows correspond to the optical microscopy images presented in Figure 6.

To validate the prevalence of using DR-NIR spectroscopy for biomass estimation of *S. platensis* cultures, and, specifically, the DR-NIR spectral response of the microalga at 1062.6 nm, another *S. platensis* culture was laboratory and axenically cultivated in larger scale, using a 30 L open-type tank. The same sampling and measurement conditions were performed, i.e., 10 mL of culture were subtracted each day of the cultivation, and their DR-NIR spectrum was acquired. It should be noted that after each measurement, the sample could be returned to the culture, ensuring that sterile conditions are followed during sampling and measurement. The spectral response at 1062.6 nm, as derived from PCA for the various cultivation days of *S. platensis*, is shown in Figure 5. The growth estimation results are similar to previous findings, following an exponential pattern, which is typical for algal cultures [2,67].

**Figure 6:**
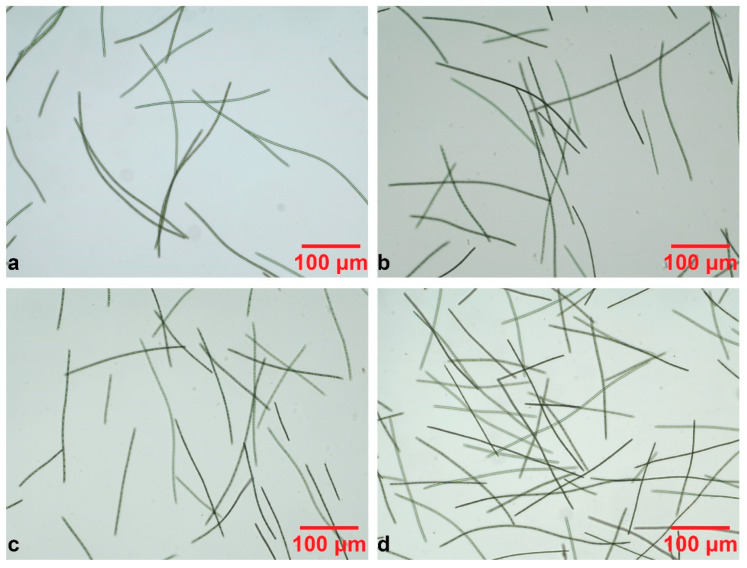
The representative optical microscopy images of the 9-day culture of *S. platensis*: (**a**) 0; (**b**) 2nd; (**c**) 3rd; and (**d**) 9th day of cultivation.

Figure 6 displays representative images obtained by optical microscopy of the same *S. platensis* culture, cultivated in a 30 L open-type tank for 9 days. In general, the optical images support the results presented in Figure 5, as the entire cultivation period exhibits the exponential growth rate of *S. platensis*. Apart from the initial and the last day of cultivation (Figure 6a and Figure 6d, respectively), days 2 (Figure 6b) and 3 (Figure 6c) are also presented, as they show a similar concentration of filaments, which supports the findings of the spectral response as presented in Figure 5. The microscopic images show that the microalgae filaments are linear, their length is not stable, which agrees with the literature [10,26,27].

## 4. Discussion

NIR spectroscopy in diffuse reflectance mode was applied to evaluate its ability to follow *S. platensis* cultivation growth, without any sample preparation, i.e., without washing out the culture medium, while using simple and well-known statistical analysis. The DR-NIR spectral response at 1062.6 nm of *S. platensis* exhibits linearity with its biomass content and the results were further validated by estimating the *S. platensis* daily growth rate in a large-scale (30 L) cultivation.

The results show that NIR measurements can be used for the growth rate biomass estimation of *S. platensis*, without eliminating the influence of the culture medium. It should be noted that although the culture medium is considered to be consumed during the cultivation period of *S. platensis* [68], and the present study was performed with freshly prepared SM, the results from the 30 L open-type tank validate the analysis, as the spectral response follows the typical exponential biomass growth rate of microalgae. It should also be noted that microalgae biomass does not remain stable during its cultivation in terms of quality. While in the present study, laboratory conditions were followed, contamination during field conditions should be taken into consideration. Furthermore, cell death that leads to the presence of cellular and extracellular components that are left behind, which is something that applies both to laboratory and field condition cultivation should be factored in. Following this, and to ensure the consistency of the results for pilot-scale or industrial-scale cultivations, the quality of the culture should be checked using optical microscopy, and each laboratory should create its own internal calibration curve, as different cultivation media may be used for *S. platensis* (e.g., Zarrouk’s media).

The findings indicate that the method can be used as an alternative to determine the growth of *S. platensis*, with the advantages of being fast and inexpensive in terms of measurement, and not time consuming in terms of sample preparation, i.e., removing the cultivation medium with successive washing and filtration cycles. It should be noted that the method essentially does not decrease the culture’s biomass because, after the measurement, during which the sample remains intact, the sample can be returned. Following this, DR-NIR spectroscopy is considered as a prominent method for direct measurements of *S. platensis* cultivations, for online monitoring, with the appropriate setup, allowing for in situ observations without sampling.

## Figures and Tables

**Table 1:** The Stock Solutions (SLs) components for the preparation of Spirulina Medium that was used for the cultivation of *S. platensis* [57].

Stock Solution SL	Component	Stock Solution SL	Component
SL I	NaHCO_3_ (13.61 g)	SL III	ZnSO_4_·7H_2_O (1 mL of 1 g/L)
(500 mL)	Na_2_CO_3_ (4.03 g)	(900 mL)	MnSO_4_·4H_2_O (2 mL of 1 g/L)
	K_2_HPO_4_ (0.50 g)		H_3_BO_3_ (5 mL of 2 g/L)
	Distilled water		Co(NO_3_)_2_·6H_2_O (5 mL of 0.2 g/L)
SL II	NaNO_3_ (2.50 g)		Na_2_MoO_4_·2H_2_O (5 mL of 0.2 g/L)
(500 mL)	K_2_SO_4_ (1.00 g)		CuSO_4_·5H_2_O (1 mL of 0.005 g/L)
	NaCl (1.00 g)		Na_2_-EDTA (Titriplex III) (0.40 g)
	MgSO_4_·7H_2_O (0.20 g)		Distilled water
	CaCl_2_·2H_2_O (0.04 g)	SL IV	FeSO_4_·7H_2_O (0.70 g)
	FeSO_4_·7H_2_O (0.01 g)	(100 mL)	Na_2_-EDTA (Titriplex III) (0.40 g)
	Na_2_-EDTA (Titriplex III) (0.08 g)		Distilled water
	SL III and SL IV (5 mL)		
	Distilled water		

**Table 2:** The samples obtained by the application of the modified serial dilution protocol.

Calibration Standard	Dense *S. platensis* Culture (mL)	Spirulina Medium (mL)
10	10	0
9	9	1
8	8	2
7	7	3
6	6	4
5	5	5
4	4	6
3	3	7
2	2	8
1	1	9
0	0	10

## Data Availability

The original contributions presented in the study are included in the article, further inquiries can be directed to the corresponding author.
